# Elevated CA 125 level in a mucinous cystadenoma and a teratoma: a case report

**DOI:** 10.1186/s13256-020-02458-x

**Published:** 2020-09-03

**Authors:** Chanil Deshan Ekanayake, Nayoman Munasinghe, Iranthi Kumarasinghe, Sachini Rasnayake

**Affiliations:** 1 Department of Clinical Sciences, Faculty of Medicine, General Sir John Kotelawala Defence University, Ratmalana, Sri Lanka; 2 Department of Paraclinical Sciences, Faculty of Medicine, General Sir John Kotelawala Defence University, Ratmalana, Sri Lanka

**Keywords:** CA 125, Mature cystic teratoma, Mucinous cystadenoma

## Abstract

**Background:**

The presence of a suspicious ovarian cyst with elevated cancer antigen 125 level in a woman of reproductive age poses a serious therapeutic dilemma. Mature cystic teratomas and mucinous cystadenomas may also cause an increase in cancer antigen 125.

**Case presentation:**

A 43-year-old Sinhalese woman with a history of anovulatory subfertility for 5 years presented with heavy menstrual bleeding and secondary dysmenorrhea of 6 months’ duration. Imaging (pelvic ultrasound and computed tomography of the abdomen and pelvis) revealed a hemorrhagic cyst (6 × 4 cm) on the right side and a multilocular cyst with solid areas (10 × 7 cm) on the left side. Her cancer antigen 125 level was 2715 U/ml. Following a multidisciplinary team meeting, a fertility-sparing staging laparotomy was performed, which included right cystectomy, left oophorectomy, infracolic omentectomy, and peritoneal washings. Histology revealed a mucinous cystadenoma of the right ovary and a mature cystic teratoma on the left ovary. No malignant cells were observed in peritoneal washings. The patient’s cancer antigen 125 level dropped to 74.8 U/ml 1 month after surgery.

**Conclusion:**

Rarely, teratomas and mucinous cystadenomas may also give rise to an extremely high cancer antigen 125 level. The risk of malignancy index and risk of malignancy algorithm may both be misleading in these instances. Therefore, multidisciplinary input, fertility-sparing surgery, and follow-up are paramount to achieve optimal treatment and patient satisfaction.

## Background

The presence of a suspicious ovarian cyst with elevated cancer antigen 125 (CA 125) level in a woman of reproductive age poses a serious therapeutic dilemma in terms of achieving optimal treatment versus fulfilling the patient’s fertility expectations. CA 125 is a high-molecular-weight glycoprotein that is derived from the coelomic epithelium and as such is found in the endometrium, peritoneum, and pericardium [1, 2]. CA 125 is commonly used as a biomarker for epithelial ovarian cancer diagnosis because it correlates with malignancy risk [3]. Teratomas constitute 15–20% of all ovarian tumors and are the commonest ovarian tumor in the reproductive age group [1]. CA 125 may be elevated in 13.5–25% of teratoma cases, but it is only mildly elevated [4, 5]. Mucinous cystadenoma is also a benign mucin-containing epithelial ovarian tumor that is usually found in middle-aged women in whom CA 125 levels can rarely be elevated [6].

## Case presentation

A 43-year-old Sinhalese woman with a history of anovulatory subfertility for 5 years presented to the gynecology clinic of the University Hospital – General Sir John Kotelawala Defence University complaining of heavy menstrual bleeding and secondary dysmenorrhea of 6 months’ duration. She had undergone treatment with clomiphene citrate for eight cycles. Previous imaging did not reveal an ovarian cyst. Her CA 125 had not been measured previously. She had no loss of appetite or loss of weight. Her past medical and psychosocial history was unremarkable. She had no family history of malignancy. On examination, she was afebrile and had a soft abdomen. Her cervix appeared normal upon speculum examination. Bimanual examination revealed that her uterus was of normal size and retroverted. She had a solid nontender adnexal mass extending from the left adnexa to the pouch of Douglas.

Imaging (pelvic ultrasound and computed tomography of her abdomen and pelvis) revealed a hemorrhagic cyst (6 × 4 cm) on the right side and a multilocular cyst with solid areas (10 × 7 cm) on the left side. No peritoneal deposits and ascites were observed. The patient’s CA 125 level was 2715 U/ml. The case was discussed at a multidisciplinary team (MDT) meeting due to the patient’s history of subfertility and elevated CA 125 level.

**Fig. 1:**
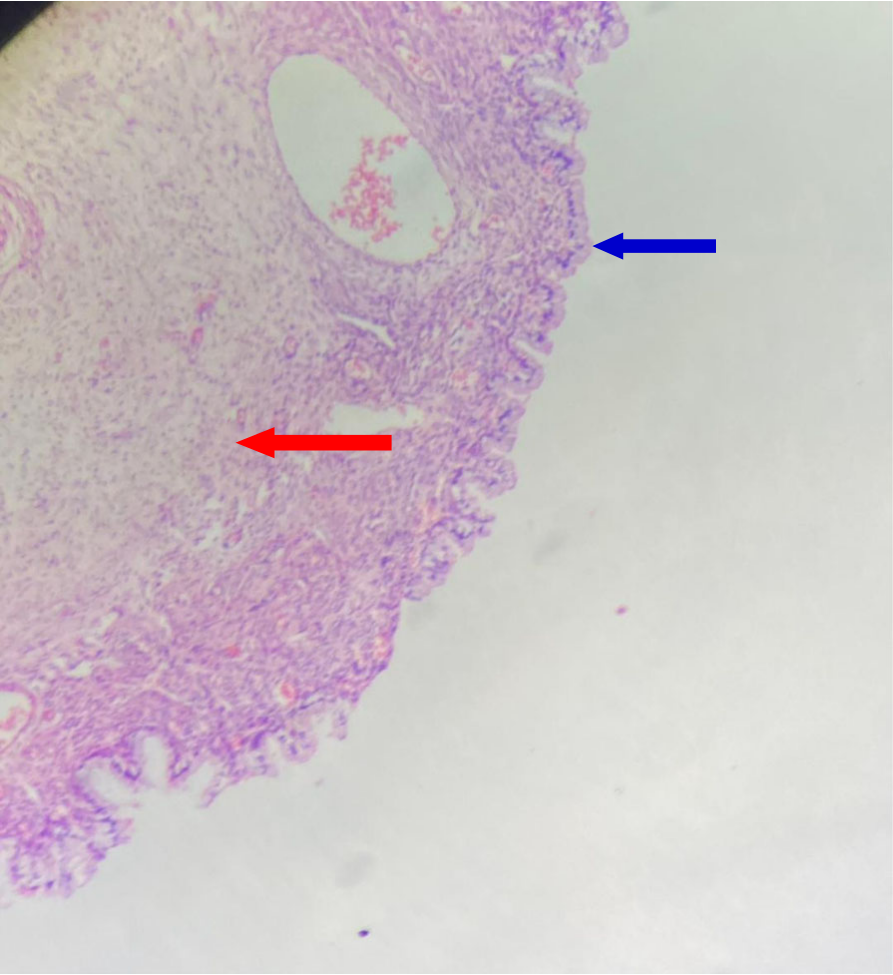
Histology of the mucinous cystadenoma in the right ovary. Blue arrow denotes columnar mucinous epithelium, red arrow shows the fibrous cyst wall

**Fig. 2:**
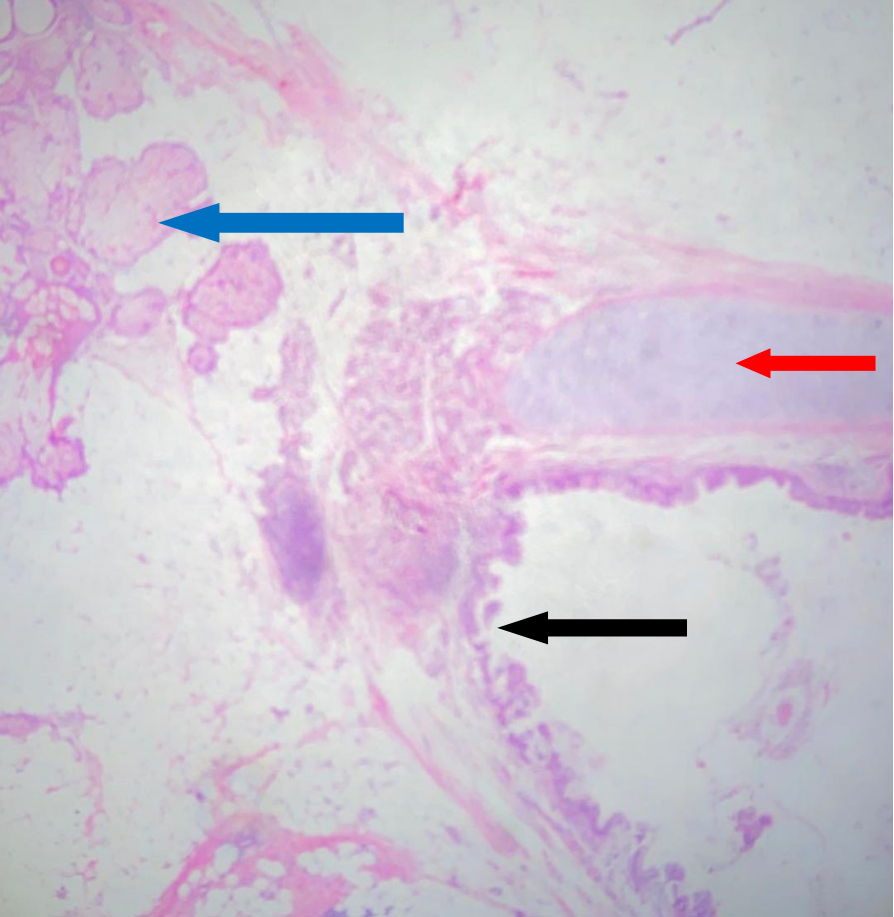
Histology of the mature cystic teratoma in the left ovary. Blue arrow shows sebaceous glands (ectoderm), red arrow shows cartilage (mesoderm) and the black arrow denotes pseudo stratified columnar ciliated epithelium (endoderm)

**Fig. 3:**
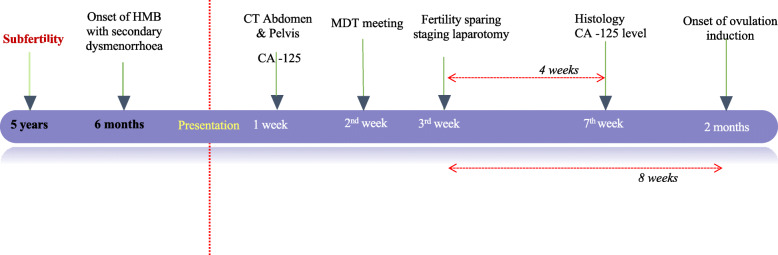
Timeline of events

A fertility-sparing staging laparotomy was performed, which included right cystectomy, left oophorectomy, infracolic omentectomy, and peritoneal washings. Histology revealed a mucinous cystadenoma on the right ovary (Fig. 1) and a mature cystic teratoma on the left ovary (Fig. 2). No malignant cells were observed in peritoneal washings. Following surgery, the patient’s CA 125 level dropped to 74.8 U/ml 1 month after surgery. She is currently being seen in follow-up and is receiving letrozole for ovulation induction. A timeline of events is shown in Fig. 3.

## Discussion

The preoperative differential diagnosis of our patient considered an epithelial ovarian cancer, endometrioma, and teratoma. An early-stage epithelial ovarian cancer was considered on the basis of the imaging findings and CA 125 level (risk of malignancy index [RMI] = 1 × 3 × 2715 = 8145). However, the patient’s long-standing history of subfertility, dysmenorrhea, and heavy menstrual bleeding suggested endometriosis. Nonetheless, the endometrioma could not explain the solid area on the left ovary. Therefore, it was more likely that there was a double pathology with an endometrioma on the right ovary and a teratoma on the left ovary.

Following an MDT, a fertility-sparing staging laparotomy was undertaken with surgical input involving an oncologic surgeon, which was in keeping with current recommendations [7]. Measurement of other tumor markers lactate dehydrogenase, alpha-fetoprotein, β-human chorionic gonadotropin, and CA 19-9 was not done because of financial limitations. The decision not to perform hysterectomy and bilateral oophorectomy was made in consideration of her fertility wishes with a planned repeat surgery depending on the histology. In hindsight, it appeared to be the correct decision because it gave her a chance of conceiving. The other pertinent question was the decision for oophorectomy on the left side, which was performed because there was minimal normal ovarian tissue.

Although it is uncommon to find an elevated CA 125 level in mature cystic teratoma, it can be mildly elevated in 13.5–25% of cases [5]. CA 125 can be found to be elevated in mucinous cystadenoma as well [6]. In our patient’s case, it may have been a combination of these two factors that may have given rise to an elevated CA 125 level, but there were no reported cases in which a teratoma or a mucinous cystadenoma accounted for such a high CA 125 level. More notably, CA 125 can be elevated in other malignancies and also in physiological and benign conditions (for example, endometriosis, uterine fibroids, pelvic inflammatory disease) [1]. Exorbitantly high CA 125 levels similar to the value seen in our patient’s case have been reported in leaking endometriomas [2].

This diagnostic dilemma due to CA 125 is further compounded because the RMI and risk of malignancy algorithm (ROMA) place too much emphasis on the CA 125 level [8]. Thus, for these reasons, the RMI and ROMA can be provide unreliable estimates of the risk of malignancy in women of reproductive age.

## Conclusion

Rarely, teratomas and mucinous cystadenomas may also give rise to extremely high CA 125 levels, which may cause a diagnostic dilemma. Therefore, multidisciplinary input, fertility-sparing surgery, and follow-up are paramount to achieve optimal treatment and patient satisfaction.

## Data Availability

Not applicable.

## References

[1] Kumar V, Abbas AK, Aster JC. Female genital system and breast. In: Robbins basic pathology. 9th ed. Philadelphia: Elsevier; 2013. p. 698–700.

[2] Rao S, Kapurubandara S, Anpalagan A. Elevated CA 125 in a case of leaking endometrioma. Case Rep Obstet Gynecol. 2018;2018:2385048. 10.1155/2018/2385048 PMC6157201 30298110

[3] Bast RC Jr, Badgwell D, Lu Z, *et al.* New tumor markers: CA125 and beyond. Int J Gynecol Cancer. 2005;15:274–81. 10.1111/j.1525-1438.2005.00441.x 16343244

[4] Ionescu CA, Matei A, Navolan D, *et al.* Correlation of ultrasound features and the risk of ovarian malignancy algorithm score for different histopathological subtypes of benign adnexal masses. Medicine (Baltimore). 2018;97(31):e11762. 10.1097/MD.0000000000011762. PMC6081138 30075600

[5] Cho HY, Kim K, Jeon YT, Kim YB. CA19-9 elevation in ovarian mature cystic teratoma: discrimination from ovarian cancer - CA19-9 level in teratoma. Med Sci Monit. 2013;19:230–5. 10.12659/MSM.883865 PMC3628710 23539074

[6] Dong L, Cui H, Li XP, *et al.* Clinical value of serum CA19-9, CA125 and CP2 in mucinous ovarian tumor: a retrospective study of 273 patients [in Chinese]. Zhonghua Fu Chan Ke Za Zhi. 2008;43:5–8. 18366923

[7] Royal College of Obstetricians and Gynaecologists. Fertility-sparing treatments in gynaecological cancers. Scientific impact paper no. 35. 2013.

[8] Lycke M, Kristjansdottire B, Sundfeldt KA. A multicenter clinical trial validating the performance of HE4, CA125, risk of ovarian malignancy algorithm and risk of malignancy index. Gynecol Oncol. 2018;151:159–65. 10.1016/j.ygyno.2018.08.025 30149898

